# Supplementary material for: Combination of pre-adapted bacteriophage therapy and antibiotics for treatment of fracture-related infection due to pandrug-resistant Klebsiella pneumoniae
Source: Nat Commun. 2022 Jan 18;13:302. doi: 10.1038/s41467-021-27656-z (PMC8766457; doi:10.1038/s41467-021-27656-z)
Supplement: Supplementary file 2 — Description of Additional Supplementary Files [file 41467_2021_27656_MOESM2_ESM.pdf]

Description of Additional Supplementary Files

File Name: Supplementary Data 1
Description: Single nucleotide polymorphisms (SNPs) present in adapted phage M1.

File Name: Supplementary Data 2
Description: Genome islands and prophages present in the patient‟s Klebsiella pneumoniae strains.

File Name: Supplementary Data 3
Description: : Single nucleotide polymorphisms (SNPs) present in the patient‟s Klebsiella pneumoniae strains

File Name: Supplementary Data 4
Description: Annotation of antimicrobial resistance (AMR) genes present in the patient‟s Klebsiella pneumoniae strains.

File Name: Supplementary Data 5
Description: National Center for Biotechnology Information (NCBI) accession codes of phage and bacterial genome sequences
