# Supplementary material for: Combination of pre-adapted bacteriophage therapy and antibiotics for treatment of fracture-related infection due to pandrug-resistant Klebsiella pneumoniae
Source: Nat Commun. 2022 Jan 18;13:302. doi: 10.1038/s41467-021-27656-z (PMC8766457; doi:10.1038/s41467-021-27656-z)
Supplement: Supplementary file 8 — Reporting Summary [file 41467_2021_27656_MOESM8_ESM.pdf]

nature research

Corresponding author(s): NMED-BC112505

Last updated by author(s): Nov 13, 2021

Reporting Summary

Nature Research wishes to improve the reproducibility of the work that we publish. This form provides structure for consistency and transparency in reporting. For further information on Nature Research policies, see our Editorial Policies and the Editorial Policy Checklist.

Statistics

For all statistical analyses, confirm that the following items are present in the figure legend, table legend, main text, or Methods section.

n/a	Confirmed	
☐	☒	The exact sample size (*n*) for each experimental group/condition, given as a discrete number and unit of measurement
☐	☒	A statement on whether measurements were taken from distinct samples or whether the same sample was measured repeatedly
☐	☒	The statistical test(s) used AND whether they are one- or two-sided<br>*Only common tests should be described solely by name; describe more complex techniques in the Methods section.*
☒	☐	A description of all covariates tested
☒	☐	A description of any assumptions or corrections, such as tests of normality and adjustment for multiple comparisons
☐	☒	A full description of the statistical parameters including central tendency (e.g. means) or other basic estimates (e.g. regression coefficient) AND variation (e.g. standard deviation) or associated estimates of uncertainty (e.g. confidence intervals)
☐	☒	For null hypothesis testing, the test statistic (e.g. *F*, *t*, *r*) with confidence intervals, effect sizes, degrees of freedom and *P* value noted<br>*Give P values as exact values whenever suitable.*
☒	☐	For Bayesian analysis, information on the choice of priors and Markov chain Monte Carlo settings
☒	☐	For hierarchical and complex designs, identification of the appropriate level for tests and full reporting of outcomes
☒	☐	Estimates of effect sizes (e.g. Cohen's *d*, Pearson's *r*), indicating how they were calculated

Our web collection on statistics for biologists contains articles on many of the points above.

Software and code

Policy information about availability of computer code

Data collection	*Provide a description of all commercial, open source and custom code used to collect the data in this study, specifying the version used OR state that no software was used.*
Data analysis	FastQ, trimmomatic, Nanoplot, Porechop, Nanofilt, Unicycles, snippy, BLASTn, eggNOG-mapper, abricate, PHASTER, Islandviews SPAdes, MEGAX, RASTtk, BLASTp, tRNAscan-SE.

For manuscripts utilizing custom algorithms or software that are central to the research but not yet described in published literature, software must be made available to editors and reviewers. We strongly encourage code deposition in a community repository (e.g. GitHub). See the Nature Research guidelines for submitting code & software for further information.

Data

Policy information about availability of data

All manuscripts must include a data availability statement. This statement should provide the following information, where applicable:

- Accession codes, unique identifiers, or web links for publicly available datasets
- A list of figures that have associated raw data
- A description of any restrictions on data availability

With regard to Klebsiella pneumoniae genome: The annotated genome of isolate Kp36336 was submitted to the genbank database (Accession number CP066511-CP066515).
With regard to phage vB_KpnM_M1 genome: The final genbank file was submitted to the NCBI database (Accession number MW448170).
The authors declare that all other data supporting the findings of this study are available within the article and its supplementary information files.

Note: if not yet publicly available a time of potential review, genbank files may be accessed through these links:

https://dataview.ncbi.nlm.nih.gov/object/PRJNA686374?reviewer=cd63p1t77hkj0h5on6e2nt09s3
https://www.ncbi.nlm.nih.gov/nuccore/MW448170

Field-specific reporting

Please select the one below that is the best fit for your research. If you are not sure, read the appropriate sections before making your selection.

- [x] Life sciences
- [ ] Behavioural & social sciences
- [ ] Ecological, evolutionary & environmental sciences

For a reference copy of the document with all sections, see nature.com/documents/nr-reporting-summary-flat.pdf

Life sciences study design

All studies must disclose on these points even when the disclosure is negative.

Sample size	1 patient (case study)
Data exclusions	None
Replication	Experiments were repeated at least three times.
Randomization	Not applicable (case study)
Blinding	Not applicable (case study)

Reporting for specific materials, systems and methods

We require information from authors about some types of materials, experimental systems and methods used in many studies. Here, indicate whether each material, system or method listed is relevant to your study. If you are not sure if a list item applies to your research, read the appropriate section before selecting a response.

Materials & experimental systems

n/a	Involved in the study	
x		Antibodies
x		Eukaryotic cell lines
x		Palaeontology and archaeology
x		Animals and other organisms
	x	Human research participants
x		Clinical data
x		Dual use research of concern

Methods

n/a	Involved in the study	
x		ChIP-seq
x		Flow cytometry
x		MRI-based neuroimaging

Human research participants

Policy information about studies involving human research participants

Population characteristics	1 patient (case study)
Recruitment	In the context of salvage therapy
Ethics oversight	The Ethical Committee of the Erasme Hospital, Brussels, Belgium

Note that full information on the approval of the study protocol must also be provided in the manuscript.
